# Religious End-World delusion: psychopathological types

**DOI:** 10.1192/j.eurpsy.2022.1810

**Published:** 2022-09-01

**Authors:** E. Gedevani, G. Kopeiko, O. Borisova, P. Orekhova, V. Kaleda

**Affiliations:** 1 FSBSI Mental Health Research Center, Researching Group Of Specific Forms Of Mental Disorders, Moscow, Russian Federation; 2 Federal State Budgetary Scientific Institution «Mental Health Research Center», Researching Group Of Specific Forms Of Mental Disorders, Moscow, Russian Federation; 3 Mental Health Research Centre, Department Of Youth Psychatry, Moscow, Russian Federation

**Keywords:** religious delusion, End-World delusion, delusional behavior, schizophrénia

## Abstract

**Introduction:**

Diagnostics of End-World delusion with religious content (EWDRC) is relevant due to its insufficient exploration, difficulty in differential diagnostics and social danger of the delusional behavior.

**Objectives:**

To develop a typology based on psychopathological and phenomenological features.

**Methods:**

Sixty patients with EWDRC were examined. Psychopathological and statistical methods were applied.

**Results:**

Study of EWDRC found heterogeneity of clinical appearances. Two different types were identified: apocalyptic and eschatological. **The apocalyptic type** (51 patients, 85%) was characterized by prevalence of End-World ideas in an acute sensual delusion. Due to heterogeneity of delusion’s dynamics two subtypes were identified: - Subtype 1 (31 patients, 61%) was characterized with long period of development (changes in the stages) of different delusion’s types: delusion of perception, importance, staging, and the antagonistic one. Psychotic symptoms were quickly reduced with antipsychotic therapy. - Subtype 2 (20 patients, 39%) was characterized with rapid development of delusion’s stages up to oneiro-catatonic states which were hardly jugulated. **Eschatological type** (9 patients, 15%) was characterized by the systematized interpretive delusion with individual interpretation of apocalyptic signs. These states evolved within mixed forms of schizophrenia.

**Conclusions:**

The analysis of EWDRC revealed the apocalyptic type’s acute course. Patients with the apocalyptic type have a premonition of upcoming End-World, and feel themselves engaged in it. The eschatological type is based on the systematized interpretive End-World delusion with “confirmations” found in everyday life. The results showed the high risk of the delusional behavior in patients with EWDRC which requires careful approach to the diagnostics and treatment of these conditions.

**Disclosure:**

No significant relationships.

